# Supplementary figures and images for: Ras/MAPK signalling intensity defines subclonal fitness in a mouse model of hepatocellular carcinoma
Source: eLife. 2023 Jan 19;12:e76294. doi: 10.7554/eLife.76294 (PMC9891719; doi:10.7554/eLife.76294)

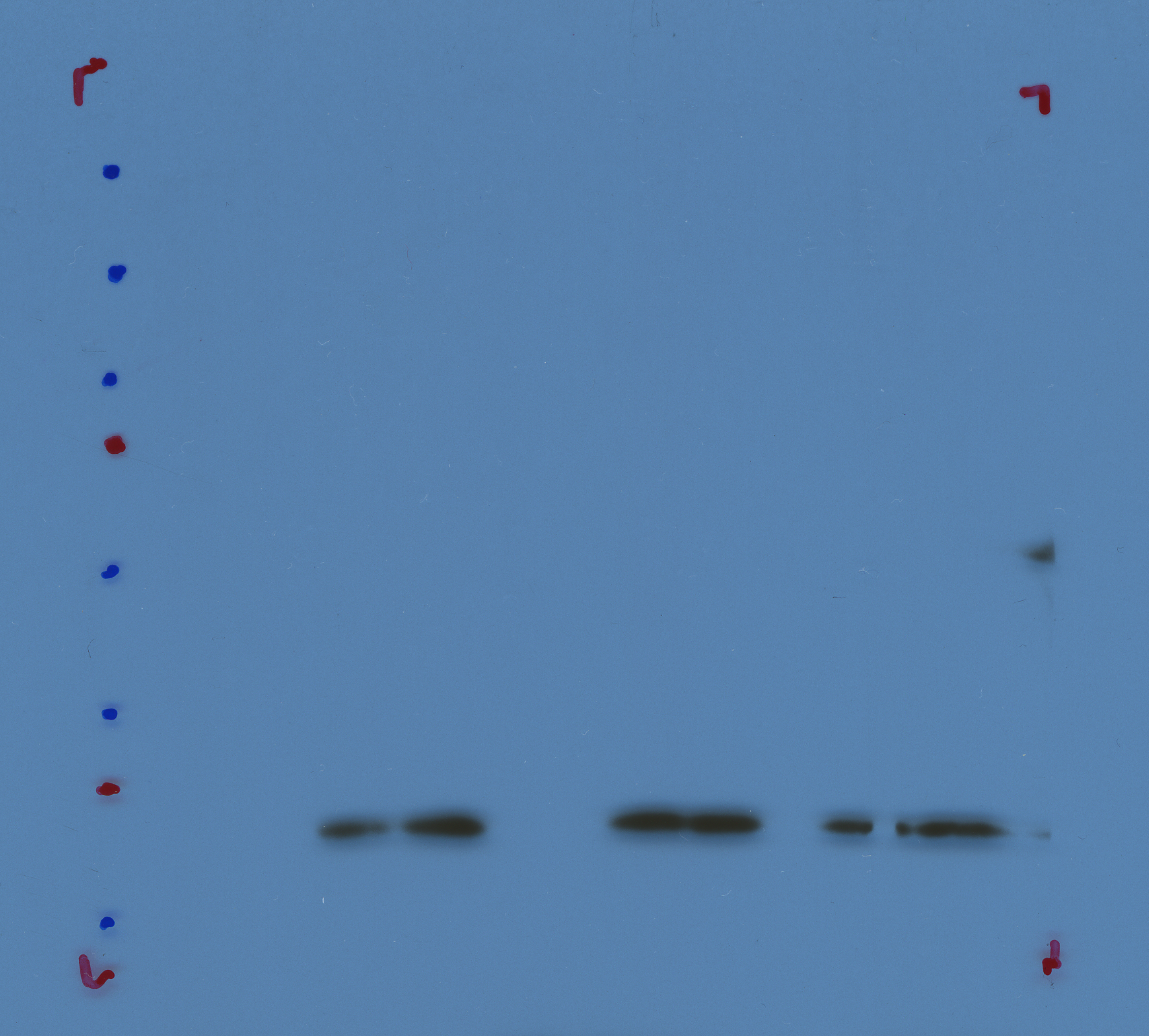

Supplement: Figure 1—source data 1. [file elife-76294-fig1-data1.zip › Figure 1-source data1/Figure 1A-source data WB-Ras .tif]

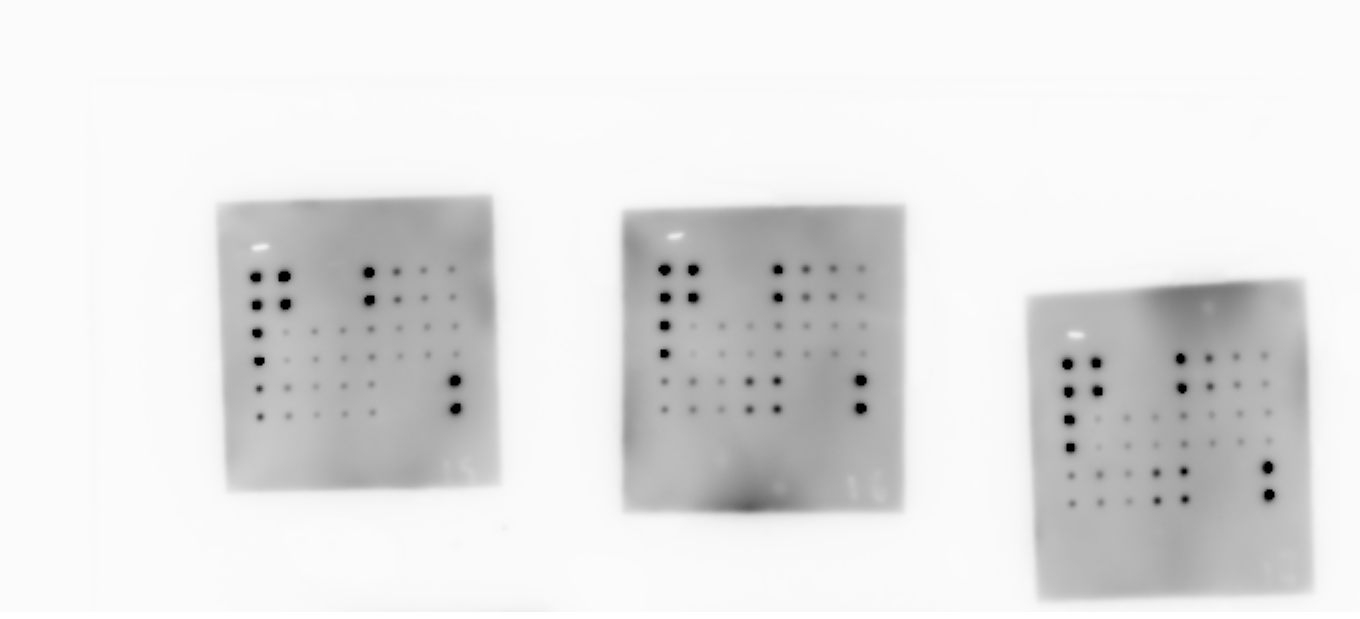

Supplement: Figure 1—source data 1. [file elife-76294-fig1-data1.zip › Figure 1-source data1/Figure 1C-source data 1.tif]

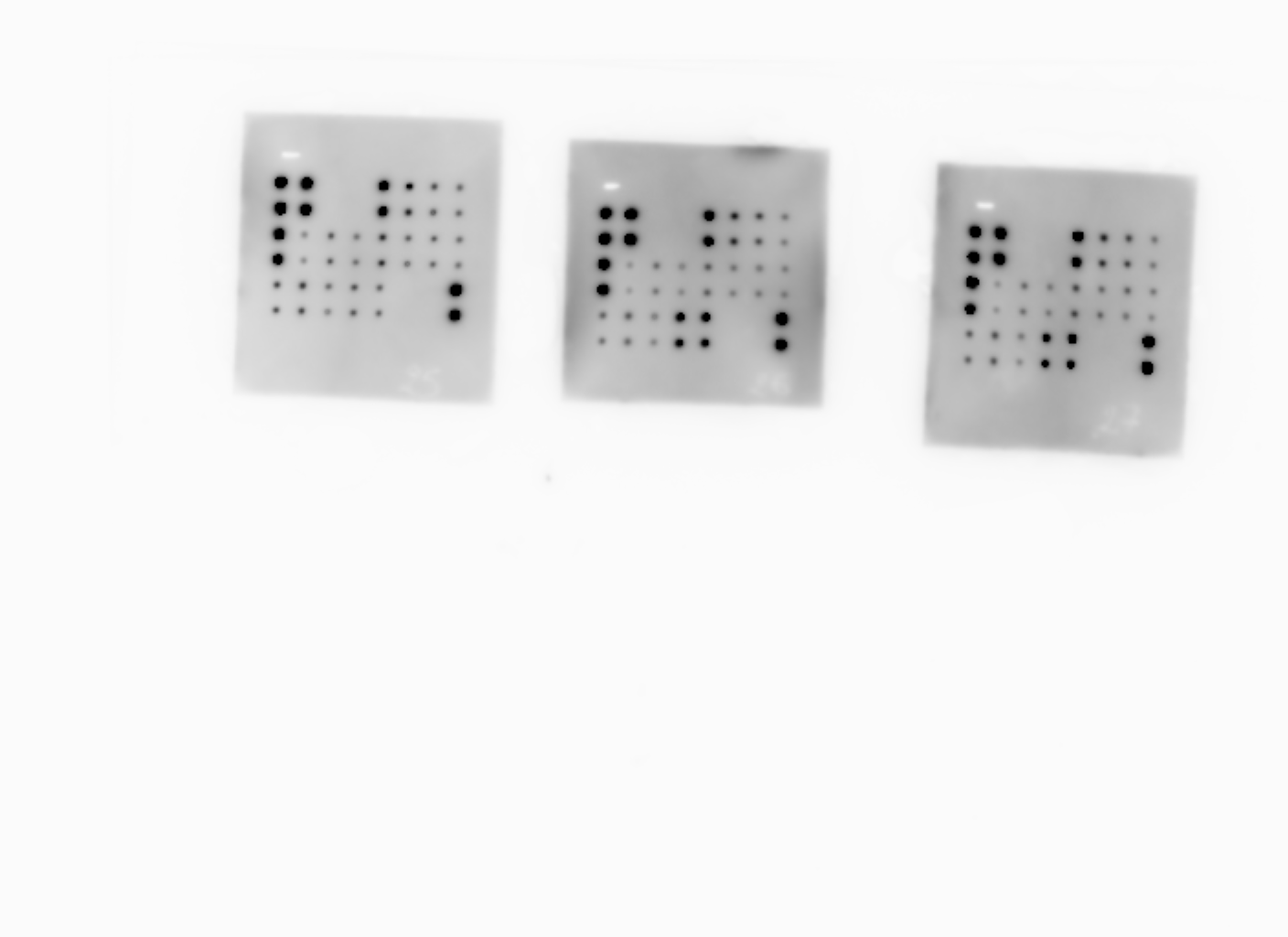

Supplement: Figure 1—source data 1. [file elife-76294-fig1-data1.zip › Figure 1-source data1/Figure1C-source data 2.tif]

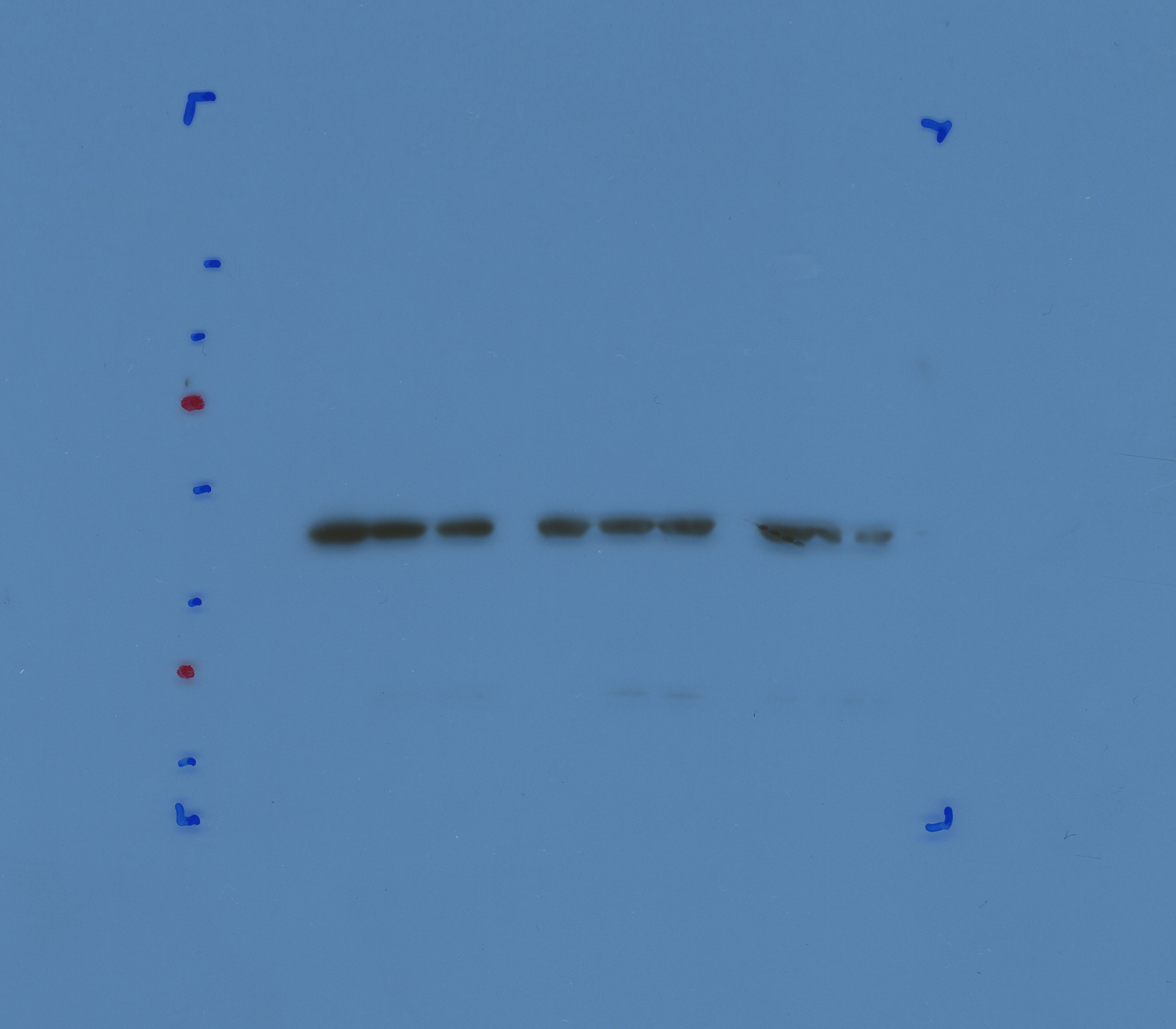

Supplement: Figure 1—source data 1. [file elife-76294-fig1-data1.zip › Figure 1-source data1/Figure 1A-source data 1 WB-actin .tif]

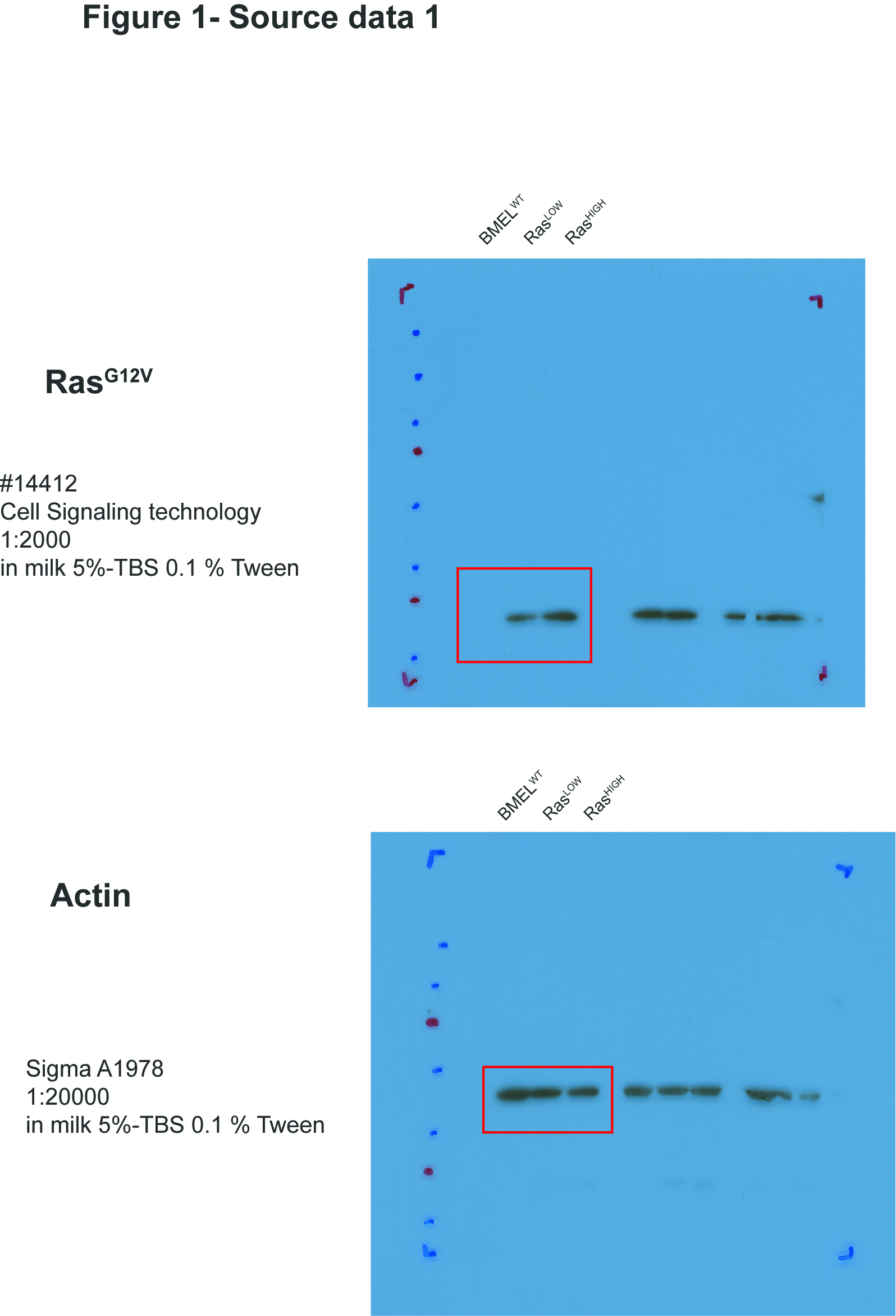

Supplement: Figure 1—source data 1. [file elife-76294-fig1-data1.zip › Figure 1-source data1/FIgure 1 source data 1.tif]
