# Supplementary material for: Ras/MAPK signalling intensity defines subclonal fitness in a mouse model of hepatocellular carcinoma
Source: eLife. 2023 Jan 19;12:e76294. doi: 10.7554/eLife.76294 (PMC9891719; doi:10.7554/eLife.76294)
Supplement: Supplementary file 1. — (a) Gene signature for the low threshold Ras/MAPK signalling (Figure 1E left panel). (b) Gene expression modified only in RasHIGH cells (Figure 1E middle panel). (c) Genes for which expression correlated with intensity of Ras/MAPK signalling (Figure 1E right panel). (d) Genes differentialy expressed in peritoneal tumour cells versus liver tumour cells. (e) GSEA identified enriched gene sets liver versus peritoneum isolated tumour cells. [file elife-76294-supp1.docx]

**Supplemetary file 1: RNAseq and gene expression data**

**Supplementary file 1a: gene signature for the low threshold Ras/MAPK signalling (Figure 1E left panel)**

| n=160 |  | WT vs Ras^LOW^ | | WT vs Ras^HIGH^ | | Ras^LOW^ vs Ras^HIGH^ | |
| --- | --- | --- | --- | --- | --- | --- | --- |
| Gene symbol | Gene id | log2FoldChange | padjExactTest | log2FoldChange | padjExactTest | log2FoldChange | padjExactTest |
| Lgals2 | *107753* | 1.51 | 4.22E-08 | 1.66 | 8.78E-10 | 0.67 | 1.00E+00 |
| Rap1b | *215449* | 3.17 | 2.44E-14 | 3.97 | 1.61E-46 | 0.01 | 3.92E-01 |
| Nup107 | *103468* | 3.59 | 3.38E-05 | 3.99 | 6.45E-06 | 0.47 | 1.00E+00 |
| Hmga1 | *15361* | 2.13 | 2.30E-07 | 2.64 | 2.63E-03 | 0.26 | 1.00E+00 |
| Rbp4 | *19662* | 2.03 | 3.40E-14 | 2.50 | 3.31E-05 | 0.15 | 1.00E+00 |
| Slc35e3 | *215436* | 3.02 | 3.70E-29 | 3.57 | 1.62E-38 | 0.09 | 1.00E+00 |
| Ankrd1 | *107765* | -1.65 | 3.59E-04 | -2.70 | 2.09E-07 | 0.01 | 3.51E-01 |
| Ccn1 | *16007* | -1.86 | 3.56E-03 | -3.06 | 2.15E-06 | 0.08 | 9.78E-01 |
| Sprr1a | *20753* | -2.23 | 5.75E-04 | -1.62 | 4.69E-02 | 0.47 | 1.00E+00 |
| Prkg2 | *19092* | 1.82 | 7.48E-05 | 2.36 | 1.85E-18 | 0.11 | 1.00E+00 |
| Phlda2 | *22113* | 2.57 | 9.07E-04 | 3.11 | 1.58E-04 | 0.32 | 1.00E+00 |
| Peg3os | *100169889* | -2.44 | 6.38E-03 | -1.71 | 2.04E-02 | 0.41 | 1.00E+00 |
| Psph | *100678* | 1.71 | 1.75E-03 | 2.19 | 3.16E-14 | 0.15 | 1.00E+00 |
| Rps6ka2 | *20112* | 2.30 | 1.64E-16 | 3.33 | 7.56E-06 | 0.02 | 4.32E-01 |
| Peg3 | *18616* | -1.77 | 5.07E-11 | -1.52 | 1.20E-08 | 0.48 | 1.00E+00 |
| Suox | *211389* | 1.77 | 1.63E-05 | 1.76 | 8.02E-10 | 0.89 | 1.00E+00 |
| Glipr1 | *73690* | -2.11 | 8.40E-03 | -2.07 | 1.47E-03 | 0.98 | 1.00E+00 |
| Cdh2 | *12558* | -1.55 | 1.64E-08 | -2.05 | 2.28E-13 | 0.11 | 1.00E+00 |
| Osgin1 | *71839* | -1.68 | 3.40E-03 | -1.50 | 6.51E-03 | 0.5 | 1.00E+00 |
| Pcsk9 | *100102* | 1.81 | 2.13E-04 | 3.02 | 8.91E-09 | 0 | 8.94E-02 |
| St3gal6 | *54613* | 2.37 | 6.04E-08 | 2.76 | 4.63E-10 | 0.25 | 1.00E+00 |
| Plekhf1 | *72287* | 1.61 | 2.49E-06 | 1.52 | 8.95E-04 | 0.7 | 1.00E+00 |
| Dusp1 | *19252* | -2.26 | 4.46E-03 | -2.51 | 1.09E-03 | 0.75 | 1.00E+00 |
| Serpinb1a | *66222* | 3.33 | 1.23E-06 | 5.05 | 2.02E-14 | 0 | 1.76E-02 |
| Pacrg | *69310* | 2.88 | 6.31E-05 | 4.36 | 5.38E-36 | 0 | 4.22E-03 |
| Gm36117 | *102639918* | 1.64 | 1.34E-04 | 2.56 | 2.33E-05 | 0.01 | 3.50E-01 |
| Ifi203 | *15950* | 2.74 | 1.31E-03 | 4.51 | 1.18E-07 | 0 | 9.47E-02 |
| Cryab | *12955* | -2.42 | 2.91E-06 | -4.03 | 9.02E-14 | 0.01 | 3.48E-01 |
| Deptor | *97998* | 2.34 | 4.19E-12 | 3.04 | 4.58E-05 | 0.12 | 1.00E+00 |
| Shf | *435684* | 1.51 | 1.48E-03 | 2.72 | 2.05E-03 | 0.03 | 6.15E-01 |
| Nes | *18008* | -2.07 | 2.18E-14 | -2.18 | 7.73E-16 | 0.81 | 1.00E+00 |
| Bicc1 | *83675* | -2.15 | 7.14E-16 | -2.98 | 8.07E-28 | 0.17 | 1.00E+00 |
| Dglucy | *217830* | -1.85 | 2.81E-09 | -2.10 | 1.61E-14 | 0.43 | 1.00E+00 |
| Aif1l | *108897* | -1.51 | 2.86E-05 | -2.38 | 7.55E-11 | 0.08 | 9.46E-01 |
| Aldh1a1 | *11668* | -2.99 | 4.16E-17 | -2.71 | 2.13E-22 | 0.78 | 1.00E+00 |
| Arl14 | *71619* | 2.50 | 5.33E-07 | 3.30 | 4.95E-17 | 0.03 | 6.68E-01 |
| Ifi202b | *26388* | 1.66 | 6.47E-05 | 2.21 | 1.32E-10 | 0.13 | 1.00E+00 |
| Csf1 | *12977* | -1.93 | 4.01E-03 | -2.83 | 1.97E-05 | 0.07 | 9.32E-01 |
| Cdc42bpg | *240505* | -1.79 | 3.01E-06 | -1.73 | 3.29E-07 | 0.9 | 1.00E+00 |
| Egfr | *13649* | -1.99 | 2.71E-13 | -2.86 | 4.62E-26 | 0.02 | 4.71E-01 |
| Bhlhb9 | *70237* | 2.04 | 1.17E-10 | 1.93 | 4.01E-10 | 0.71 | 1.00E+00 |
| Bcas1 | *76960* | 2.80 | 1.60E-10 | 3.01 | 7.32E-06 | 0.54 | 1.00E+00 |
| Dmbt1 | *12945* | 2.95 | 2.65E-08 | 4.43 | 1.59E-14 | 0 | 3.42E-03 |
| Nrg1 | *211323* | -1.71 | 4.33E-09 | -2.09 | 2.68E-14 | 0.36 | 1.00E+00 |
| Dcxr | *67880* | -2.14 | 9.29E-07 | -2.26 | 8.62E-10 | 0.84 | 1.00E+00 |
| Ak1 | *11636* | -2.20 | 1.89E-14 | -3.13 | 1.38E-28 | 0.19 | 1.00E+00 |
| Cdhr5 | *72040* | 2.20 | 7.48E-05 | 3.06 | 1.34E-16 | 0.02 | 4.98E-01 |
| Pdk1 | *228026* | 1.87 | 2.73E-03 | 2.78 | 3.43E-03 | 0.11 | 1.00E+00 |
| Slc4a11 | *269356* | -1.80 | 9.82E-04 | -2.16 | 2.62E-09 | 0.53 | 1.00E+00 |
| Fcho1 | *74015* | 2.31 | 2.07E-05 | 2.54 | 2.69E-08 | 0.53 | 1.00E+00 |
| Akap5 | *238276* | -2.90 | 4.38E-07 | -4.86 | 1.66E-15 | 0.03 | 5.65E-01 |
| Kcnh2 | *16511* | 3.17 | 6.31E-04 | 3.62 | 8.01E-03 | 0.49 | 1.00E+00 |
| Trim7 | *94089* | -1.55 | 2.82E-03 | -1.94 | 1.30E-08 | 0.58 | 1.00E+00 |
| Serpinb6b | *20708* | -1.72 | 1.91E-03 | -3.38 | 1.57E-14 | 0.08 | 9.57E-01 |
| Adgrg6 | *215798* | -1.83 | 1.26E-10 | -2.22 | 8.59E-16 | 0.4 | 1.00E+00 |
| Gsn | *227753* | -2.14 | 4.10E-11 | -3.11 | 2.73E-22 | 0.01 | 2.95E-01 |
| Gstm1 | *14862* | -1.80 | 7.27E-02 | -1.91 | 1.04E-02 | 0.89 | 1.00E+00 |
| Arid5a | *214855* | 1.94 | 4.53E-06 | 2.81 | 7.42E-15 | 0.01 | 3.38E-01 |
| Mst1r | *19882* | 2.73 | 6.72E-13 | 2.55 | 1.61E-06 | 0.63 | 1.00E+00 |
| Slc16a6 | *104681* | 1.88 | 3.02E-05 | 2.15 | 8.10E-08 | 0.4 | 1.00E+00 |
| Bscl2 | *14705* | -1.51 | 7.47E-06 | -2.35 | 8.87E-14 | 0.03 | 5.65E-01 |
| Fgd3 | *30938* | -2.53 | 2.38E-03 | -3.41 | 2.28E-05 | 0.07 | 9.23E-01 |
| Gadd45b | *17873* | -1.74 | 2.99E-07 | -3.15 | 1.47E-20 | 0 | 1.74E-01 |
| Hoxa3 | *15400* | 1.50 | 3.10E-03 | 1.83 | 5.16E-06 | 0.38 | 1.00E+00 |
| Pfkp | *56421* | 1.58 | 1.79E-04 | 2.37 | 9.77E-04 | 0.05 | 8.03E-01 |
| Tnfrsf1b | *21938* | 1.73 | 4.79E-04 | 3.53 | 1.24E-06 | 0 | 3.66E-02 |
| Foxq1 | *15220* | -1.79 | 2.82E-07 | -2.15 | 7.55E-11 | 0.42 | 1.00E+00 |
| Slco3a1 | *108116* | -1.72 | 3.89E-09 | -2.42 | 9.27E-17 | 0.04 | 7.36E-01 |
| Tns1 | *21961* | -2.55 | 3.88E-08 | -2.83 | 3.00E-10 | 0.67 | 1.00E+00 |
| Psca | *72373* | -2.53 | 1.18E-02 | -3.67 | 8.04E-05 | 0.11 | 1.00E+00 |
| Fn1 | *14268* | -3.57 | 1.19E-05 | -3.87 | 2.12E-06 | 0.77 | 1.00E+00 |
| Plxna4 | *243743* | 3.05 | 1.75E-04 | 4.22 | 3.28E-09 | 0.01 | 2.63E-01 |
| Rcor2 | *104383* | 1.56 | 6.11E-03 | 1.69 | 2.68E-04 | 0.77 | 1.00E+00 |
| Rassf9 | *237504* | -1.63 | 2.60E-03 | -1.56 | 2.73E-03 | 0.88 | 1.00E+00 |
| Glipr2 | *384009* | -2.71 | 2.59E-12 | -3.80 | 4.94E-21 | 0.03 | 6.54E-01 |
| Serpinb9b | *20706* | -2.75 | 2.20E-02 | -5.02 | 2.88E-06 | 0.16 | 1.00E+00 |
| Kank1 | *107351* | -2.96 | 1.09E-13 | -5.26 | 2.76E-32 | 0.01 | 4.08E-01 |
| Hoxb3 | *15410* | -3.35 | 2.97E-11 | -2.93 | 6.14E-11 | 0.59 | 1.00E+00 |
| Hoxb5 | *15413* | -4.81 | 4.62E-11 | -3.89 | 3.10E-10 | 0.54 | 1.00E+00 |
| Hoxb8 | *15416* | -3.43 | 2.34E-10 | -1.89 | 5.10E-05 | 0.02 | 4.51E-01 |
| Mtus1 | *102103* | -1.99 | 2.16E-08 | -3.44 | 1.11E-22 | 0.05 | 7.65E-01 |
| Ces2g | *72361* | -4.44 | 1.55E-07 | -5.99 | 1.94E-11 | 0.17 | 1.00E+00 |
| Serpinb8 | *20725* | -2.82 | 2.68E-07 | -5.47 | 3.28E-16 | 0.01 | 3.38E-01 |
| Fam149a | *212326* | -3.05 | 2.76E-05 | -5.02 | 1.49E-11 | 0.11 | 1.00E+00 |
| P3h2 | *210530* | -1.63 | 7.72E-05 | -3.20 | 2.29E-11 | 0 | 5.49E-02 |
| Zfp764 | *233893* | -2.77 | 1.59E-04 | -3.77 | 1.19E-08 | 0.32 | 1.00E+00 |
| H60b | *667281* | -3.28 | 1.76E-04 | -4.68 | 1.27E-07 | 0.43 | 1.00E+00 |
| Sorl1 | *20660* | -1.73 | 1.84E-03 | -3.07 | 2.73E-08 | 0 | 4.26E-02 |
| Ccbe1 | *320924* | -1.83 | 5.45E-02 | -3.41 | 1.51E-09 | 0.34 | 1.00E+00 |
| Htatip2 | *53415* | -4.78 | 7.44E-11 | -5.95 | 1.66E-15 | 0.62 | 1.00E+00 |
| Ces2e | *234673* | -5.68 | 1.02E-06 | -6.98 | 6.90E-09 | 0.51 | 1.00E+00 |
| Cutal | *77996* | -2.32 | 7.89E-06 | -1.85 | 5.15E-05 | 0.6 | 1.00E+00 |
| Gm9949 | *225609* | -3.19 | 1.87E-05 | -5.20 | 7.90E-12 | 0.16 | 1.00E+00 |
| Afap1l2 | *226250* | -3.98 | 3.61E-05 | -4.89 | 2.45E-07 | 0.14 | 1.00E+00 |
| Gsta3 | *14859* | -7.08 | 8.13E-05 | -8.26 | 7.37E-06 | 0 | 1.00E+00 |
| Tgfb2 | *21808* | -1.71 | 7.88E-05 | -3.69 | 6.54E-23 | 0.07 | 9.16E-01 |
| Notch3 | *18131* | -3.37 | 1.88E-04 | -4.14 | 9.88E-07 | 0.5 | 1.00E+00 |
| Samd9l | *209086* | -2.67 | 3.56E-03 | -3.57 | 2.09E-05 | 0.26 | 1.00E+00 |
| Dusp8 | *18218* | -1.52 | 3.11E-02 | -2.02 | 1.30E-03 | 0.27 | 1.00E+00 |
| Dkk2 | *56811* | -1.95 | 5.67E-02 | -3.54 | 6.67E-05 | 0.06 | 8.83E-01 |
| Wt1 | *22431* | -3.39 | 5.56E-15 | -6.26 | 1.54E-35 | 0.05 | 8.01E-01 |
| Cd59a | *12509* | -3.40 | 1.17E-10 | -5.92 | 5.37E-24 | 0.16 | 1.00E+00 |
| Mmp2 | *17390* | -2.43 | 3.46E-09 | -3.85 | 1.50E-21 | 0.06 | 8.53E-01 |
| Smarca2 | *67155* | -2.56 | 3.46E-08 | -3.47 | 5.08E-15 | 0.28 | 1.00E+00 |
| Serpina1a | *20700* | -4.41 | 1.61E-05 | -5.35 | 1.17E-07 | 0 | 1.00E+00 |
| Ctla2b | *13025* | -2.63 | 7.75E-05 | -7.35 | 5.86E-17 | 0 | 1.57E-01 |
| Pla1a | *85031* | -2.04 | 2.36E-03 | -2.23 | 1.45E-07 | 0.85 | 1.00E+00 |
| Nid2 | *18074* | -1.90 | 1.19E-02 | -4.25 | 3.86E-08 | 0 | 1.26E-01 |
| Nnat | *18111* | -3.31 | 1.85E-02 | -4.91 | 3.15E-04 | 0.44 | 1.00E+00 |
| Shank2 | *210274* | -2.67 | 4.92E-17 | -3.33 | 7.85E-27 | 0.27 | 1.00E+00 |
| Gria3 | *53623* | -5.65 | 7.64E-47 | -9.67 | 1.20E-71 | 0 | 2.12E-01 |
| Akr1c14 | *105387* | -4.51 | 1.37E-23 | -8.75 | 2.06E-43 | 0.07 | 9.12E-01 |
| Mapkapk3 | *102626* | -1.85 | 1.27E-05 | -1.74 | 9.88E-07 | 0.91 | 1.00E+00 |
| Gdpd5 | *233552* | -1.54 | 1.71E-05 | -1.92 | 4.37E-09 | 0.41 | 1.00E+00 |
| Heg1 | *77446* | -2.04 | 1.94E-04 | -1.89 | 5.97E-04 | 0.76 | 1.00E+00 |
| Spns2 | *216892* | -1.65 | 2.57E-04 | -2.59 | 6.48E-08 | 0.03 | 6.01E-01 |
| Serpinb9 | *20723* | -1.71 | 5.69E-04 | -3.27 | 1.62E-21 | 0.16 | 1.00E+00 |
| Rnf130 | *59044* | -3.83 | 1.07E-03 | -6.65 | 3.31E-07 | 0.07 | 9.09E-01 |
| Rab38 | *72433* | -4.45 | 3.76E-02 | -5.48 | 4.75E-03 | 0.59 | 1.00E+00 |
| Ccn4 | *22402* | -1.90 | 3.56E-02 | -5.04 | 3.74E-11 | 0.04 | 7.37E-01 |
| Nectin1 | *58235* | -2.14 | 1.45E-11 | -2.95 | 4.20E-21 | 0.04 | 7.16E-01 |
| Mab21l4 | *71874* | -2.49 | 3.09E-11 | -4.16 | 3.68E-28 | 0.1 | 1.00E+00 |
| Pik3ap1 | *83490* | -2.12 | 1.45E-09 | -3.31 | 8.85E-24 | 0.05 | 7.65E-01 |
| Ckmt1 | *12716* | -3.03 | 2.22E-05 | -4.51 | 6.43E-10 | 0.15 | 1.00E+00 |
| Cox6b2 | *333182* | -3.34 | 3.03E-03 | -5.08 | 6.53E-06 | 0.42 | 1.00E+00 |
| Tmem14a | *75712* | -1.70 | 2.89E-03 | -2.98 | 1.28E-09 | 0.1 | 1.00E+00 |
| Def6 | *23853* | -2.41 | 3.77E-03 | -2.95 | 2.29E-04 | 0.34 | 1.00E+00 |
| Sytl1 | *269589* | -3.71 | 5.84E-03 | -4.71 | 3.02E-04 | 0.31 | 1.00E+00 |
| Siglecg | *243958* | -3.91 | 5.77E-03 | -6.30 | 9.92E-06 | 0.01 | 3.75E-01 |
| Tap1 | *21354* | -3.06 | 3.82E-16 | -3.96 | 2.05E-26 | 0.23 | 1.00E+00 |
| Tbc1d8 | *54610* | -2.32 | 6.68E-12 | -3.35 | 4.28E-24 | 0.05 | 7.66E-01 |
| Ctla2a | *13024* | -2.82 | 2.53E-08 | -5.17 | 1.37E-21 | 0.01 | 2.62E-01 |
| Ly6e | *17069* | -3.96 | 9.60E-05 | -5.84 | 1.38E-08 | 0.2 | 1.00E+00 |
| 9230114K14Rik | *414108* | -1.63 | 9.00E-05 | -2.48 | 2.53E-11 | 0.06 | 8.97E-01 |
| Hoxb7 | *15415* | -4.78 | 2.42E-20 | -5.08 | 1.16E-24 | 0.79 | 1.00E+00 |
| Hoxb6 | *15414* | -3.91 | 2.62E-16 | -3.53 | 3.96E-16 | 0.64 | 1.00E+00 |
| Serpina1b | *20701* | -5.09 | 7.17E-13 | -5.18 | 1.05E-13 | 0.97 | 1.00E+00 |
| Wnt9a | *216795* | -3.56 | 7.22E-10 | -6.78 | 4.63E-23 | 0 | 1.53E-01 |
| Mcam | *84004* | -4.11 | 4.04E-07 | -5.67 | 3.63E-11 | 0.05 | 8.01E-01 |
| Cd109 | *235505* | -1.68 | 3.59E-04 | -3.17 | 1.63E-10 | 0.01 | 2.28E-01 |
| Pros1 | *19128* | -1.96 | 9.33E-03 | -2.45 | 4.32E-04 | 0.23 | 1.00E+00 |
| Inka2 | *109050* | -1.68 | 1.99E-07 | -2.77 | 5.43E-20 | 0.02 | 4.52E-01 |
| Dlc1 | *50768* | -4.75 | 7.63E-05 | -8.52 | 3.05E-10 | 0.09 | 1.00E+00 |
| Gstm2 | *14863* | -1.96 | 6.77E-03 | -2.91 | 3.93E-06 | 0.24 | 1.00E+00 |
| Fas | *14102* | -3.94 | 2.55E-17 | -5.91 | 5.90E-32 | 0.26 | 1.00E+00 |
| Gm38426 | *100503676* | -2.14 | 6.67E-05 | -2.35 | 1.96E-06 | 0.7 | 1.00E+00 |
| Celf5 | *319586* | -2.96 | 2.45E-03 | -3.71 | 2.89E-05 | 0.41 | 1.00E+00 |
| Bmyc | *107771* | -2.59 | 3.77E-03 | -5.34 | 2.28E-07 | 0.01 | 2.74E-01 |
| Tubb4a | *22153* | -4.07 | 1.30E-02 | -3.97 | 7.68E-03 | 0.85 | 1.00E+00 |
| Pclaf | *68026* | -1.73 | 1.86E-02 | -3.90 | 1.69E-09 | 0.04 | 7.40E-01 |
| Scx | *20289* | -2.28 | 4.96E-08 | -4.29 | 1.67E-24 | 0.15 | 1.00E+00 |
| Creb3l1 | *26427* | -1.90 | 5.46E-02 | -2.86 | 1.50E-05 | 0.44 | 1.00E+00 |
| Ano3 | *228432* | -2.86 | 3.40E-16 | -4.72 | 8.69E-48 | 0.09 | 1.00E+00 |
| Ddit4l | *73284* | -3.35 | 5.16E-24 | -6.24 | 1.98E-60 | 0 | 1.99E-01 |
| Arhgef26 | *622434* | -1.53 | 2.97E-03 | -5.32 | 3.60E-38 | 0 | 1.04E-01 |
| Foxj1 | *15223* | -2.87 | 6.40E-21 | -4.84 | 2.96E-50 | 0.09 | 1.00E+00 |
| 1700007K13Rik | *69327* | -4.53 | 4.18E-08 | -6.63 | 3.42E-13 | 0.08 | 9.47E-01 |
| Cdh6 | *12563* | -2.48 | 4.04E-03 | -3.71 | 1.03E-05 | 0.1 | 1.00E+00 |
| Ndn | *17984* | -6.93 | 2.59E-06 | -9.82 | 2.56E-09 | 0.19 | 1.00E+00 |
| Igfbp7 | *29817* | -4.02 | 7.18E-44 | -8.11 | 2.39E-111 | 0 | 9.92E-02 |

**Supplementary file 1b: gene expression modified only in RasHIGH cells (Figure 1E middle panel)**

| n=65 |  | Ras Low vs WT | | Ras High vs WT | | Ras High vs Ras Low | |
| --- | --- | --- | --- | --- | --- | --- | --- |
| Gene symbol | Gene id | log2FoldChange | padjExactTest | log2FoldChange | padjExactTest | log2FoldChange | padjExactTest |
| Prl2c2 | *18811* | 2.68 | 7.54E-01 | 6.57 | 1.80E-19 | 3.88 | 1.88E-08 |
| Anxa13 | *69787* | 0.38 | 9.24E-01 | 2.52 | 4.21E-21 | 2.14 | 1.40E-07 |
| Apoc2 | *11813* | 1.40 | 9.63E-01 | 5.46 | 5.51E-12 | 4.05 | 5.14E-07 |
| Prl2c5 | *107849* | 2.80 | 7.34E-01 | 6.00 | 5.40E-43 | 3.19 | 8.57E-12 |
| Il1rn | *16181* | 1.30 | 9.13E-01 | 5.57 | 5.12E-47 | 4.25 | 6.47E-28 |
| Sord | *20322* | 1.42 | 1.14E-01 | 3.32 | 4.39E-09 | 1.89 | 2.26E-03 |
| Arap3 | *106952* | 2.42 | 1.21E-01 | 4.46 | 1.86E-54 | 2.03 | 3.47E-07 |
| Mrc1 | *17533* | 1.69 | 2.03E-01 | 3.33 | 2.18E-25 | 1.63 | 4.38E-04 |
| Glrp1 | *14659* | 1.62 | 1.79E-01 | 3.31 | 4.21E-13 | 1.69 | 3.70E-03 |
| Pla2g7 | *27226* | 1.53 | 1.31E-01 | 3.63 | 1.59E-16 | 2.10 | 6.71E-06 |
| Kcnn4 | *16534* | 2.57 | 2.70E-01 | 5.27 | 8.53E-19 | 2.69 | 3.61E-06 |
| Mndal | *100040462* | 2.28 | 2.74E-02 | 4.35 | 4.34E-10 | 2.06 | 5.53E-03 |
| Tm4sf1 | *17112* | 2.15 | 3.35E-02 | 6.32 | 1.66E-15 | 4.17 | 1.79E-07 |
| Rhox5 | *18617* | 2.24 | 2.04E-02 | 3.96 | 2.16E-15 | 1.72 | 5.33E-03 |
| Sigirr | *24058* | 0.90 | 9.53E-01 | 3.30 | 7.25E-13 | 2.39 | 9.59E-04 |
| Ugt1a7c | *394432* | -1.56 | 7.69E-01 | 1.90 | 6.56E-03 | 3.45 | 7.18E-08 |
| Syt7 | *54525* | 2.20 | 1.96E-01 | 4.51 | 1.92E-48 | 2.30 | 1.88E-08 |
| Dab2 | *13132* | -0.48 | 3.33E-01 | -2.23 | 3.80E-14 | -1.75 | 1.80E-05 |
| Selp | *20344* | 3.30 | 3.84E-02 | 5.14 | 2.30E-43 | 1.84 | 1.07E-05 |
| Usp43 | *216835* | 1.26 | 2.57E-02 | 3.02 | 8.41E-21 | 1.76 | 1.17E-04 |
| Inpp5j | *170835* | 0.75 | 6.85E-01 | 2.93 | 1.35E-13 | 2.17 | 4.60E-07 |
| Tmem173 | *72512* | -0.40 | 8.50E-01 | 1.51 | 2.20E-03 | 1.90 | 1.75E-03 |
| Havcr2 | *171285* | 1.55 | 5.38E-01 | 5.51 | 1.38E-16 | 3.95 | 2.79E-10 |
| Steap3 | *68428* | 1.69 | 9.16E-02 | 4.51 | 3.25E-07 | 2.81 | 3.08E-03 |
| Csn3 | *12994* | 0.03 | 1.00E+00 | 3.57 | 1.10E-03 | 3.53 | 9.32E-03 |
| Kif5c | *16574* | -0.40 | 8.36E-01 | 2.75 | 8.93E-12 | 3.14 | 7.52E-14 |
| Map3k6 | *53608* | 0.21 | 1.00E+00 | 1.98 | 8.61E-09 | 1.76 | 3.25E-04 |
| Rgl1 | *19731* | -0.40 | 8.25E-01 | 1.94 | 1.70E-06 | 2.34 | 2.29E-07 |
| Slco4a1 | *108115* | -2.68 | 2.06E-02 | 1.52 | 7.33E-03 | 4.19 | 6.41E-10 |
| Spaca9 | *69987* | 0.11 | 1.00E+00 | 2.80 | 1.73E-04 | 2.68 | 5.53E-03 |
| Tmem252 | *226040* | -0.62 | 9.74E-01 | 3.94 | 2.59E-08 | 4.56 | 3.67E-08 |
| Trim14 | *74735* | 0.46 | 7.36E-01 | 2.30 | 4.87E-07 | 1.84 | 3.71E-04 |
| Epdr1 | *105298* | -0.84 | 2.24E-02 | -2.46 | 6.01E-17 | -1.62 | 6.91E-04 |
| Clrn3 | *212070* | 0.64 | 8.81E-01 | 2.43 | 5.45E-09 | 1.79 | 1.72E-03 |
| Cyth4 | *72318* | -0.23 | 1.00E+00 | 1.61 | 2.62E-05 | 1.83 | 1.01E-03 |
| Naip1 | *17940* | 0.56 | 8.48E-01 | 3.27 | 2.33E-08 | 2.71 | 1.72E-05 |
| Qrfp | *227717* | -0.23 | 9.93E-01 | 2.10 | 2.81E-03 | 2.33 | 7.75E-03 |
| Sh3bp2 | *24055* | -0.09 | 1.00E+00 | 1.59 | 1.73E-03 | 1.68 | 4.02E-03 |
| Hs6st2 | *50786* | -0.94 | 3.58E-03 | -3.79 | 1.10E-39 | -2.84 | 1.13E-12 |
| Cd53 | *12508* | 2.52 | 7.52E-02 | 5.45 | 3.21E-09 | 2.92 | 1.32E-03 |
| Ceacam1 | *26365* | 1.98 | 7.19E-01 | 5.52 | 2.13E-07 | 3.53 | 2.25E-03 |
| Sec16b | *89867* | 0.10 | 9.99E-01 | 2.46 | 5.00E-09 | 2.36 | 9.91E-07 |
| Slc14a1 | *108052* | 2.47 | 8.55E-01 | 5.27 | 1.25E-21 | 2.79 | 1.34E-06 |
| Tnxb | *81877* | 1.97 | 1.86E-01 | 3.62 | 2.49E-26 | 1.65 | 8.74E-04 |
| Map2 | *17756* | -0.07 | 1.00E+00 | -2.60 | 5.94E-04 | -2.53 | 4.07E-07 |
| Shroom3 | *27428* | 0.22 | 9.56E-01 | -1.72 | 4.47E-08 | -1.94 | 6.60E-03 |
| Them6 | *223626* | -1.31 | 1.08E-03 | -2.99 | 2.01E-16 | -1.68 | 1.19E-02 |
| 9330188P03Rik | *380930* | 3.10 | 8.04E-01 | 6.74 | 5.79E-11 | 3.64 | 8.60E-05 |
| Cdh5 | *12562* | 4.04 | 5.34E-01 | 6.87 | 1.02E-20 | 2.82 | 2.95E-05 |
| Tlr4 | *21898* | 0.97 | 2.55E-01 | 2.96 | 2.21E-09 | 1.98 | 4.42E-05 |
| Pla2g16 | *225845* | -1.35 | 4.76E-02 | -3.44 | 4.05E-07 | -2.09 | 4.09E-03 |
| Lrp2 | *14725* | -1.30 | 3.90E-01 | -4.23 | 3.25E-04 | -2.92 | 5.92E-02 |
| Pcsk5 | *18552* | -2.53 | 5.80E-02 | -5.90 | 1.35E-05 | -3.37 | 3.54E-02 |
| Cep126 | *234915* | -0.75 | 2.81E-01 | -2.41 | 4.91E-09 | -1.66 | 1.82E-02 |
| Zfp185 | *22673* | -0.85 | 1.43E-01 | -2.45 | 2.21E-09 | -1.60 | 3.62E-02 |
| Fgfr3 | *14184* | -1.37 | 9.86E-04 | -3.89 | 1.65E-18 | -2.51 | 3.32E-03 |
| Upk3b | *100647* | -2.20 | 7.06E-02 | -5.67 | 2.55E-06 | -3.46 | 5.88E-02 |
| Ssbp2 | *66970* | -0.84 | 1.33E-01 | -3.37 | 4.40E-17 | -2.53 | 5.50E-02 |
| Pkia | *18767* | -1.25 | 4.79E-04 | -3.46 | 1.26E-24 | -2.21 | 1.72E-05 |
| Epb41l3 | *13823* | -1.82 | 3.52E-01 | -5.45 | 1.13E-03 | -3.63 | 4.40E-09 |
| Mpzl2 | *14012* | -0.39 | 7.42E-01 | -2.62 | 6.99E-06 | -2.23 | 1.11E-06 |
| Npr2 | *230103* | -0.82 | 1.66E-01 | -3.21 | 5.52E-18 | -2.40 | 2.85E-03 |
| Fgf18 | *14172* | -0.81 | 3.04E-01 | -3.87 | 4.64E-17 | -3.05 | 5.68E-02 |
| Uchl1 | *22223* | -0.95 | 5.12E-01 | -3.57 | 8.49E-05 | -2.61 | 9.20E-03 |
| Nuak1 | *77976* | -2.44 | 1.08E-01 | -3.95 | 3.42E-03 | -1.52 | 1.44E-02 |

**Supplementary file 1c: genes for which expression correlated with intensity of Ras/MAPK signalling (Figure 1E right panel)**

| n=15 |  | Ras Low vs WT | | Ras High vs WT | | Ras High vs Ras Low | |
| --- | --- | --- | --- | --- | --- | --- | --- |
| Gene symbol | Gene id | log2FoldChange | padjExactTest | log2FoldChange | padjExactTest | log2FoldChange | padjExactTest |
| AI467606 | *101602* | 2.43 | 1.95E-05 | 4.24 | 1.57E-25 | 1.80 | 6.28E-05 |
| Aim2 | *383619* | 1.79 | 5.59E-03 | 3.97 | 2.60E-36 | 2.18 | 3.47E-07 |
| Dynap | *75577* | 5.84 | 4.86E-03 | 7.92 | 2.35E-97 | 2.08 | 3.43E-07 |
| Htra3 | *78558* | 1.86 | 1.49E-03 | 3.74 | 8.65E-09 | 1.87 | 5.89E-03 |
| Itgb7 | *16421* | 5.26 | 3.29E-04 | 6.92 | 1.15E-47 | 1.66 | 3.16E-03 |
| Tspan13 | *66109* | 1.93 | 8.38E-04 | 3.88 | 7.00E-10 | 1.95 | 2.28E-03 |
| Ppp2r2b | *72930* | -2.89 | 2.31E-20 | -8.11 | 8.07E-79 | -5.23 | 8.11E-03 |
| Cbr1 | *12408* | -2.44 | 9.54E-03 | -5.23 | 2.47E-07 | -2.80 | 4.13E-03 |
| Pmp22 | *18858* | -2.35 | 3.06E-03 | -4.56 | 5.31E-08 | -2.21 | 7.67E-03 |
| Ptp4a3 | *19245* | -4.01 | 1.85E-41 | -8.04 | 4.59E-103 | -4.03 | 4.29E-02 |
| Pmaip1 | *58801* | -2.16 | 1.09E-13 | -4.45 | 1.01E-47 | -2.29 | 4.12E-07 |
| Thbs1 | *21825* | -3.56 | 5.11E-05 | -5.42 | 3.05E-09 | -1.86 | 4.97E-05 |
| Akap12 | *83397* | -5.27 | 3.74E-11 | -8.60 | 7.60E-22 | -3.33 | 5.98E-02 |
| Sulf2 | *72043* | -2.18 | 1.65E-15 | -4.61 | 1.04E-56 | -2.43 | 1.45E-09 |
| Crip2 | *68337* | -2.45 | 3.10E-05 | -4.07 | 8.05E-11 | -1.62 | 1.63E-04 |

**Supplementary file 1d: genes differentialy expressed in peritoneal tumor cells vs liver tumor cells**

log2 fold change >1; p<0.05

| Gene symbol | log2FoldChange | p-value |
| --- | --- | --- |
| Fgg | Inf | 1.05E-06 |
| Fga | Inf | 3.81E-06 |
| Clec4f | Inf | 2.61E-07 |
| Mug1 | Inf | 1.04E-05 |
| Serpina3k | Inf | 3.42E-04 |
| Clec4g | Inf | 3.74E-05 |
| Saa1 | Inf | 4.97E-03 |
| C8a | Inf | 5.12E-03 |
| Hrg | Inf | 5.94E-03 |
| Mat1a | Inf | 2.90E-02 |
| Fgl1 | 6.51 | 1.39E-03 |
| Glp1r | 4.07 | 2.25E-12 |
| Prss12 | 3.13 | 6.29E-03 |
| Ahsg | 6.18 | 2.10E-06 |
| Cited1 | 2.97 | 3.58E-04 |
| Csn3 | 2.92 | 2.65E-19 |
| Pzp | 2.83 | 4.27E-03 |
| Alb | 6.09 | 1.39E-04 |
| Kctd15 | 2.66 | 1.02E-02 |
| Fgb | 5.65 | 1.90E-05 |
| Tmem74b | 2.54 | 4.54E-05 |
| Npr3 | 2.45 | 1.40E-04 |
| Cdh23 | 2.40 | 4.58E-03 |
| Itih4 | 5.54 | 3.57E-05 |
| Aplp1 | 2.25 | 1.54E-10 |
| Gipr | 2.12 | 3.32E-24 |
| Padi1 | 2.04 | 8.66E-11 |
| Ceacam1 | 1.91 | 1.54E-06 |
| Tmprss11b | 1.91 | 2.08E-07 |
| Sh3gl3 | 1.79 | 3.40E-05 |
| Map3k9 | 1.76 | 2.52E-03 |
| Klhl3 | 1.71 | 7.81E-09 |
| Tmod1 | 1.69 | 1.21E-05 |
| Rnf152 | 1.69 | 2.43E-02 |
| Lpar1 | 1.66 | 3.89E-17 |
| Mgarp | 1.64 | 5.12E-09 |
| Dhrs3 | 1.61 | 5.15E-07 |
| Hsd11b1 | 1.60 | 3.05E-05 |
| Tmprss11e | 1.56 | 2.22E-17 |
| Apba1 | 1.55 | 9.39E-09 |
| Sox12 | 1.47 | 6.86E-03 |
| P2ry12 | 1.37 | 2.31E-06 |
| Adgra2 | 1.36 | 5.36E-05 |
| G0s2 | 1.34 | 1.28E-06 |
| Fyn | 1.33 | 6.46E-14 |
| Olr1 | 1.31 | 3.74E-07 |
| Nmnat2 | 1.30 | 6.71E-04 |
| Clic5 | 1.29 | 5.93E-04 |
| Lncenc1 | 1.27 | 6.87E-05 |
| Kif21b | 1.27 | 9.95E-10 |
| Pi15 | 1.26 | 4.90E-04 |
| Tmem252 | 1.25 | 7.88E-08 |
| Has3 | 1.15 | 5.31E-06 |
| Selp | 1.14 | 1.42E-05 |
| Irx2 | 1.12 | 1.11E-04 |
| F2rl2 | 1.11 | 9.74E-04 |
| Fam71f2 | 1.09 | 9.05E-04 |
| Mboat2 | 1.08 | 3.84E-07 |
| Rundc3b | 1.05 | 2.63E-03 |
| Slc39a8 | 1.04 | 5.82E-03 |
| Lamb3 | 1.02 | 7.02E-09 |
| Pcolce | -1.01 | 7.15E-06 |
| Clec2d | -1.02 | 1.01E-04 |
| Slc22a18 | -1.03 | 3.14E-06 |
| Daglb | -1.03 | 9.31E-04 |
| Cercam | -1.04 | 1.09E-03 |
| Cgn | -1.04 | 1.14E-03 |
| Cfb | -1.05 | 9.75E-05 |
| Cyp27a1 | -1.08 | 2.15E-05 |
| Foxj1 | -1.08 | 2.04E-03 |
| Apoc2 | -1.09 | 3.92E-03 |
| Gstm1 | -1.09 | 1.43E-03 |
| Adamtsl5 | -1.10 | 2.98E-06 |
| Sparc | -1.10 | 8.87E-05 |
| Ildr2 | -1.11 | 5.45E-03 |
| AW112010 | -1.12 | 4.92E-03 |
| Gcnt3 | -1.12 | 5.05E-08 |
| Als2cl | -1.13 | 5.30E-12 |
| Camsap3 | -1.14 | 1.98E-05 |
| Ltbp2 | -1.17 | 7.64E-04 |
| Psrc1 | -1.19 | 2.19E-08 |
| C2 | -1.21 | 8.61E-04 |
| Cbs | -1.24 | 9.02E-03 |
| Uba7 | -1.25 | 7.85E-04 |
| Cidec | -1.39 | 7.22E-03 |
| Lgals4 | -1.40 | 7.44E-03 |
| Apob | -1.45 | 6.72E-06 |
| Sema3f | -1.49 | 5.34E-08 |
| Cideb | -1.52 | 1.98E-05 |
| Plcxd3 | -1.66 | 7.08E-04 |
| Bcas1 | -1.76 | 1.27E-03 |
| Syt8 | -1.77 | 1.73E-16 |
| Gjb1 | -1.78 | 1.02E-03 |
| Trim71 | -1.80 | 7.74E-03 |
| Mamld1 | -1.80 | 3.61E-03 |
| Col5a1 | -1.83 | 1.77E-02 |
| Igfbp6 | -1.85 | 2.97E-02 |
| Tnc | -1.94 | 8.74E-03 |
| Cdhr5 | -1.96 | 7.91E-08 |
| Tnni2 | -1.99 | 7.60E-04 |
| Pla1a | -2.01 | 1.82E-03 |
| Col15a1 | -2.10 | 2.17E-02 |
| Clrn3 | -2.15 | 4.13E-06 |
| Col5a3 | -2.16 | 1.32E-02 |
| Exoc3l4 | -2.17 | 2.70E-04 |
| Tnfaip6 | -2.22 | 3.00E-02 |
| Misp | -2.28 | 9.05E-41 |
| Cspg4 | -2.41 | 4.11E-03 |
| Mrc2 | -2.41 | 2.52E-02 |
| Spon1 | -2.60 | 1.78E-02 |
| Pdgfrb | -2.67 | 2.56E-02 |
| Col16a1 | -2.69 | 2.63E-02 |
| Acta2 | -2.75 | 1.80E-03 |
| Serpine2 | -2.83 | 4.63E-02 |
| Gas1 | -2.83 | 1.22E-02 |
| Has1 | -3.05 | 1.17E-02 |
| Col1a1 | -3.07 | 1.47E-02 |
| Mmp2 | -3.08 | 2.79E-02 |
| Serpinf1 | -3.09 | 4.06E-02 |
| Olfml3 | -3.15 | 1.23E-02 |
| Col1a2 | -3.24 | 2.24E-02 |
| Col5a2 | -3.31 | 3.24E-02 |
| Srpx2 | -3.47 | 3.63E-02 |
| Smoc2 | -3.65 | 9.59E-03 |
| Col12a1 | -3.76 | 9.76E-08 |
| Ppef1 | -3.83 | 4.72E-02 |
| Sod3 | -3.89 | 1.66E-02 |
| Loxl1 | -3.97 | 2.09E-02 |
| Thy1 | -4.10 | 2.29E-03 |
| Cthrc1 | -4.12 | 1.40E-03 |
| Fbn1 | -4.21 | 4.00E-02 |
| Cxcl12 | -4.23 | 4.13E-02 |
| Col3a1 | -4.24 | 4.22E-02 |
| Col8a1 | -4.41 | 2.48E-03 |
| Fndc1 | -4.75 | 3.01E-02 |
| Sfrp1 | -4.77 | 2.62E-02 |
| Thbs2 | -4.87 | 4.67E-04 |
| Mfap2 | -4.96 | 5.56E-04 |
| Eln | -4.98 | 4.59E-02 |
| Postn | -5.32 | 9.48E-03 |
| Cilp | -5.49 | 1.72E-02 |
| Mfap5 | -5.60 | 1.45E-02 |
| Mfap4 | -6.03 | 1.88E-02 |
| Abi3bp | -6.49 | 2.96E-03 |
| Fmod | -6.73 | 1.18E-02 |
| Svep1 | -7.27 | 1.92E-02 |
| Islr | -7.52 | 9.66E-03 |
| Tpsb2 | neg inf | 2.21E-02 |
| Ptx3 | neg inf | 4.81E-02 |
| Mmrn1 | neg inf | 2.70E-02 |
| C1qtnf3 | neg inf | 1.72E-03 |
| Moxd1 | neg inf | 1.85E-03 |
| Olfml2a | neg inf | 2.91E-03 |
| Ms4a4d | neg inf | 2.05E-02 |
| Adamts16 | neg inf | 2.36E-03 |
| Angptl1 | neg inf | 3.44E-02 |
| C1qtnf9 | neg inf | 2.62E-02 |

| **Supplementary file 1e: GSEA identified enriched gene sets liver vs peritoneum isolated tumor cells** |
| --- |
| n=27 (25 enriched in liver, 2 in peritoneal tumors) |

p-value < 0.01 and FDR < 0.1

| NAME | SIZE | ES | NES | NOM p-val | FDR q-val | FWER p-val | RANK AT MAX |
| --- | --- | --- | --- | --- | --- | --- | --- |
| GO_POSITIVE_REGULATION_OF_DEFENSE_RESPONSE | 16 | 0.925 | 1.90 | 0.003 | 0.044 | 0.033 | 12 |
| GO_POSITIVE_REGULATION_OF_PHOSPHORUS_METABOLIC_PROCESS | 38 | 0.834 | 1.71 | 0.000 | 0.090 | 0.278 | 12 |
| GO_REGULATION_OF_APOPTOTIC_SIGNALING_PATHWAY | 18 | 0.870 | 1.82 | 0.000 | 0.081 | 0.297 | 12 |
| GO_APOPTOTIC_SIGNALING_PATHWAY | 25 | 0.852 | 1.82 | 0.003 | 0.070 | 0.331 | 12 |
| GO_POSITIVE_REGULATION_OF_PROTEIN_METABOLIC_PROCESS | 51 | 0.773 | 1.82 | 0.000 | 0.063 | 0.335 | 12 |
| GO_POSITIVE_REGULATION_OF_INTRACELLULAR_SIGNAL_TRANSDUCTION | 43 | 0.800 | 1.80 | 0.000 | 0.060 | 0.405 | 12 |
| GO_POSITIVE_REGULATION_OF_PROTEIN_MODIFICATION_PROCESS | 39 | 0.839 | 1.80 | 0.000 | 0.057 | 0.416 | 12 |
| GO_REGULATION_OF_SYSTEM_PROCESS | 26 | 0.825 | 1.80 | 0.006 | 0.061 | 0.458 | 15 |
| GO_SIGNAL_TRANSDUCTION_BY_PROTEIN_PHOSPHORYLATION | 40 | 0.805 | 1.78 | 0.000 | 0.073 | 0.568 | 12 |
| GO_IMMUNE_RESPONSE_REGULATING_SIGNALING_PATHWAY | 17 | 0.888 | 1.78 | 0.006 | 0.071 | 0.583 | 12 |
| GO_REGULATION_OF_MAPK_CASCADE | 36 | 0.814 | 1.78 | 0.000 | 0.074 | 0.615 | 12 |
| GO_POSITIVE_REGULATION_OF_ESTABLISHMENT_OF_PROTEIN_LOCALIZATION | 17 | 0.872 | 1.77 | 0.009 | 0.074 | 0.639 | 12 |
| GO_REGULATION_OF_DEFENSE_RESPONSE | 27 | 0.822 | 1.77 | 0.003 | 0.073 | 0.651 | 12 |
| GO_REGULATION_OF_PROTEIN_MODIFICATION_PROCESS | 66 | 0.741 | 1.77 | 0.000 | 0.076 | 0.694 | 12 |
| GO_NEGATIVE_REGULATION_OF_CELL_DEATH | 35 | 0.789 | 1.76 | 0.006 | 0.085 | 0.747 | 17 |
| GO_POSITIVE_REGULATION_OF_CELL_DEVELOPMENT | 27 | 0.820 | 1.75 | 0.008 | 0.088 | 0.777 | 12 |
| GO_PLATELET_ACTIVATION | 20 | 0.862 | 1.74 | 0.006 | 0.089 | 0.805 | 12 |
| GO_POST_TRANSLATIONAL_PROTEIN_MODIFICATION | 19 | 0.870 | 1.74 | 0.006 | 0.087 | 0.807 | 9 |
| GO_NEGATIVE_REGULATION_OF_RESPONSE_TO_EXTERNAL_STIMULUS | 25 | 0.827 | 1.73 | 0.006 | 0.093 | 0.857 | 12 |
| GO_APOPTOTIC_PROCESS | 68 | 0.709 | 1.73 | 0.000 | 0.093 | 0.868 | 35 |
| GO_PEPTIDE_HORMONE_SECRETION | 17 | 0.863 | 1.72 | 0.009 | 0.094 | 0.913 | 15 |
| GO_REGULATION_OF_PROTEIN_LOCALIZATION | 37 | 0.765 | 1.72 | 0.003 | 0.087 | 0.916 | 15 |
| GO_REGULATION_OF_PHOSPHORUS_METABOLIC_PROCESS | 67 | 0.728 | 1.71 | 0.000 | 0.091 | 0.934 | 12 |
| GO_PROTEIN_PHOSPHORYLATION | 68 | 0.729 | 1.71 | 0.000 | 0.089 | 0.934 | 12 |
| GO_REGULATION_OF_BODY_FLUID_LEVELS | 39 | 0.769 | 1.71 | 0.008 | 0.090 | 0.94 | 27 |
| **Enriched in peritoneum** |  |  |  |  |  |  |  |
| GO_EXTRACELLULAR_MATRIX_COMPONENT | 22 | -0.817 | -1.84 | 0.000 | 0.016 | 0.02 | 87 |
| GO_COLLAGEN_TRIMER | 16 | -0.862 | -1.81 | 0.000 | 0.023 | 0.055 | 87 |
